# Topic-Aware Summarization of Lived Health Care Experiences: Large Language Model Evaluation Study

**DOI:** 10.2196/85960

**Published:** 2026-06-11

**Authors:** Maneesh Bilalpur, Megan E Hamm, Young Ji Lee, Natasha G Norman, Kathleen M Mctigue, Yanshan Wang

**Affiliations:** 1 Intelligent Systems Program, University of Pittsburgh, 4200 Fifth Avenue, Pittsburgh, PA, 15260, United States, 1 4123832712; 2 Department of Medicine, University of Pittsburgh, Pittsburgh, PA, United States; 3 School of Nursing, University of Pittsburgh, Pittsburgh, PA, United States; 4 Department of Biomedical Informatics, University of Pittsburgh, Pittsburgh, PA, United States; 5 Department of Health Information Management, University of Pittsburgh, Pittsburgh, PA, United States

**Keywords:** topic modeling, text summarization, health disparities, unstructured qualitative data, large language models, natural language processing

## Abstract

**Background:**

Existing work to understand adults’ health care experiences has focused on the analysis of patient feedback provided as written responses to after-visit surveys or social media discourse. Often, such written feedback has been studied using natural language processing techniques, such as topic detection and sentiment analysis, to provide coarse-grained insights. Storytelling is a powerful form of communication and may provide insights into factors contributing to gaps in health care outcomes and avenues for improvement. In addition, studying health care experiences using natural language processing techniques has been limited to patients. The experiences of stakeholders, such as caregivers and health care providers, remain underexplored.

**Objective:**

We extract fine-grained insights from health care experiences through narratives collected from patients, caregivers, and health care providers using large language models (LLMs). Topic detection, together with hierarchical summarization of long-form stories from individuals, offers fine-grained insights. Furthermore, the study demonstrates that generated summaries can be evaluated using the LLM-as-a-judge framework and validates the outcomes through comparisons with 2 domain experts.

**Methods:**

Fifty automatically transcribed stories of African American experiences were used to identify topics in their experiences using the latent Dirichlet allocation (LDA) technique. Stories about a given topic were summarized using an open-source LLM-based hierarchical summarization approach. Topic summaries were generated by summarizing across story summaries for each story that addressed a given topic. The generated topic summaries were rated for fabrication, accuracy, comprehensiveness, and usefulness by the GPT-4 model; its reliability was validated against the original story summaries by 2 domain experts.

**Results:**

Whisper-based automatic transcription of audio narrations achieved a Levenshtein score of 6%. Twenty-six topics were identified using LDA and labeled using the LLM in the 50 African American stories. The GPT-4 ratings suggest that topic summaries were free from fabrication, highly accurate, comprehensive, and useful. The reliability of GPT ratings compared to expert assessments showed moderate-to-high agreement (Bennett *S*-score of 0.65 or higher). Our approach identified African American experience-relevant topics, such as health behaviors, interactions with medical team members, caregiving, and symptom management, among others. Such insights could help researchers learn from unstructured datasets in an efficient manner—leveraging the communicative power of storytelling.

**Conclusions:**

The use of LDA and LLMs to identify and summarize the experiences of African American individuals suggests a variety of possible avenues for health research and possible clinical improvements to support patients and caregivers, thereby improving health outcomes.

## Introduction

### Motivation

Storytelling is a narrative style that captures information beyond objective facts by catering to the beliefs and emotions of both the narrator and the audience. Sharing lived experiences with others through stories can overcome the limitations caused by extreme objectivity in alternatives such as didactic communication [1,2]. Storytelling can successfully share health knowledge [1-4], potentially providing insight into how illness shapes people’s lives, promoting behavior change, and suggesting avenues for improving health care [5-11]. Such stories—sharing the topics deemed most important by the storyteller—can suggest innovative avenues for interventions to improve the health care ecosystem. Hence, there is a need for the health informatics community to investigate narratives of health care experiences.

Despite the efficacy of narratives in health care, their usage poses certain challenges. Information stored as oral recordings is less accessible and searchable than structured data. Furthermore, moving from a single anecdote to the derivation of common themes can be expensive and time-consuming. The use of natural language processing (NLP) techniques may help to overcome these challenges. Prior studies have examined structured feedback from patients [12], free-text feedback from social media [13], and electronic health records [14] to identify themes or topics using NLP techniques. Often, topic detection has been paired with sentiment analysis to infer an individual’s sentiments toward the topic. Such solutions offer automated approaches for understanding limitations in health care environments, the efficacy of treatment approaches, and overall experiences in patient care to provide tailored solutions [12]. However, existing literature has largely focused on structured, written-language data, with relatively limited exploration of unstructured sources such as spoken-dialogue narratives.

Spoken-dialogue narratives present a unique set of challenges; they are syntactically and semantically different from written language [15], are often lengthy, and tend to cover multiple topics within a single dialogue. Under such conditions, topic-specific sentiment analysis becomes challenging due to the lack of defined topic boundaries in the data. Summarization approaches address this limitation and are also more informative, as they provide fine-grained analysis compared to single sentiment labels derived from conventional sentiment analysis. The recent rapid expansion in applications of LLMs has demonstrated that they excel at summarizing documents across domains (including health care [16]) and have shown their efficacy in understanding spoken language [17]. Thus, they offer an effective solution for summarizing spoken dialogue. Despite their ability to process complex patterns, LLMs are constrained by their input context length. For example, the popular open-source LLM, Llama 3.2, has an input context length of 128K tokens. However, topics tend to cover multiple stories whose lengths often exceed the fixed context length of LLMs. In addition, long-form inputs (commonly encountered in spoken-dialogue narrations) pose a challenge, as they run the risk of forgetting intermediate portions of the document [18]. On the other hand, classical methods are not susceptible to such limitations, especially during inference. In this work, we evaluate the capabilities of LLMs in long-form dialogue understanding by leveraging the advantages of classical methods in topic detection to build a topic-aware summarization model for understanding spoken-dialogue narratives about health care experiences.

In this work, spoken narratives of experiences (referred to as “stories”) from the African American population are analyzed to identify underlying topics and summarize issues raised about the storytellers’ lived health care experiences. The stories are a subset of those stored in the MyPaTH Story Booth archive (termed Story Booth). We focus on stories by African American storytellers because, compared to White individuals in the United States, African Americans experience worse health outcomes and are less likely to receive health care services [19-26], despite numerous efforts to close such gaps [26-28]. Additionally, storytelling may be a particularly appealing avenue for understanding African Americans’ health experiences since the community has a strong oral tradition [4,9,10] and may also foster trust-building [24].

We focus on using NLP techniques on stories of health care experiences to identify possible factors contributing to gaps in health care delivery systems, as well as potential avenues for intervention. Storytellers include patients, caregivers, and health care professionals. Our solution offers insights through fine-grained summaries per topic, compared to existing works that are limited to coarse sentiment outcomes. Topic detection was performed over transcriptions of the long-form spoken-dialogue dataset using latent Dirichlet analysis. The detected topics were augmented with topic labels for clinical interpretability using LLMs. Furthermore, we introduce topic-aware summarization, a hierarchical approach to generate topic summaries through summaries of individual stories by topic (termed topic story summary), extracting the nature of the health care experience by each topic from stories, as depicted in the topic story summaries, using LLM-based summarization.

### Background

Attempts to develop automated solutions for topic detection and sentiment analysis often involve manual annotations. One such approach [29] found appointment access and wait times, empathy, explanation, friendliness, practice environment, and overall experiences as frequent topics in patient feedback. Similarly, another study [14] analyzed electronic health records to identify goals of care in patient care. Topic detection was used to identify suicide profiles from 300,000 decedents [30]. Researchers found that suicide profiles broadly covered 5 classes: mental health and substance problems, mental health problems, crisis, alcohol-related, and intimate partner problems, physical health problems, and polysubstance problems. Furthermore, they found demographic shifts in the suicide profiles with time showing an evolving landscape of health care needs. A recent review [12] of NLP techniques to understand patient-experience feedback compared literature in terms of machine learning techniques (supervised vs unsupervised) and data collection approaches (social media vs structured surveys). Among supervised approaches, the Naïve Bayes classifier was the best performing, while the unsupervised latent Dirichlet allocation (LDA) model was the popular alternative for topic detection. In addition to sentiment detection from human-annotated labels of patient comments, affordable annotation approaches were recently explored [31]. Using LLMs for annotating patient comments revealed that despite their ability to detect in zero-shot and few-shot settings, LLMs underperformed compared to human annotators.

A recent survey [32] confirmed that most existing literature on understanding patient experience is limited to topic detection and sentiment analysis of written feedback. The ease of data collection through the structured written language has led to its popularity in understanding health care experience feedback. Despite its advantages, the use of narrative-based approaches has been limited [32-34]. One study examined supervised models for topic detection in patient-doctor conversations [35]. The researchers used turn-level manual annotations as a gold-standard reference. They varied the input context length for the supervised models and found that topic detection improves with an increase in input context length. Summaries of spoken-dialogue medical conversations between patients and doctors were studied [36] for commercial applications such as note-taking. This line of work, which focuses on summarizing patient-doctor conversations, has been gaining attention in NLP and informatics communities [36,37]. We build upon this work by shifting the focus to the health care experiences of adults, shared through storytelling. Our narratives are focused on the quality of clinical interactions, their outcomes, and the broader impact on the day-to-day lives of patients, providers, and caregivers to identify potential causes and areas of health care disparity.

Our work leverages fine-grained details in participants’ conversational style data about their health care experiences. We believe that such details offer a nuanced understanding of problems in the health care experiences of marginalized communities. This is achieved by using a topic-aware hierarchical summarization approach to health care feedback. In the following sections, we describe the dataset, techniques for topic detection, and hierarchical summarization, and present the findings about health care experiences from the generated summaries.

## Methods

### Dataset

The MyPaTH Story Booth [38] archive is a collection of individual experiences, told from the perspective of a patient, caregiver, or health care provider. Developed as community engagement infrastructure for the PaTH Clinical Research Network, the archive includes over 1500 stories related to experiences with illness, efforts to maintain health, and interactions with health care systems [39]. Story recordings were conducted in-person or by telephone. Participants can opt to share an unstructured story or to answer prompts selected from a preapproved list. They are asked to limit their stories to 20 minutes in length or less. Funding for the MyPaTH Story Booth project was awarded in 2015, and recruitment began in March 2016. Between March 24, 2016, and August 8, 2024, 1266 stories were collected by the University of Pittsburgh Story Booth team; 167 stories were provided by African American participants (n=167, 13.2%). As of February 6, 2026, 1537 stories had been recorded in the archive.

The dataset used in this work is limited to 1120 stories—the full set collected by the University of Pittsburgh staff and fully processed at the outset of this study. From this set, 50 stories from African American participants were randomly selected for summarization and analysis. OpenAI’s Whisper model [40] was used for diarization (speaker detection and transcription) of the audio recordings. Due to the limited involvement of the interviewers, we focused only on the participants’ statements. We validated the Whisper transcriptions against manual transcriptions from the 50 validation stories to observe 35,030 out of 565,372 character-level changes (insertions, deletions, or substitutions), that is, a Levenshtein distance [41] of 6%. Manual inspection found that the diarization quality was satisfactory and that differences in labeling conventions between manual and automatic Whisper approaches were the major source of diarization errors.

### Ethical Considerations

The University of Pittsburgh’s Human Research Protection Office reviewed this study (protocols STUDY19020307 and STUDY20110315) and approved it, with a determination of no greater than minimal risk.

Stories in the database are collected with informed consent and recorded as audio files. For privacy, storytellers are instructed to avoid naming people or places. Recorded stories are reviewed, and specific identifiers (eg, names and places) are redacted, but it is possible that voices could be recognized. Narratives are included on the public Story Booth website only with the storytellers’ permission. When stories are elicited to learn about the perspectives of a specific population, storytellers are compensated at the rate of US $100 per story. Compensation policies have evolved over time, and earlier stories were collected without compensation.

### Analysis

#### Overview

**Figure 1.**
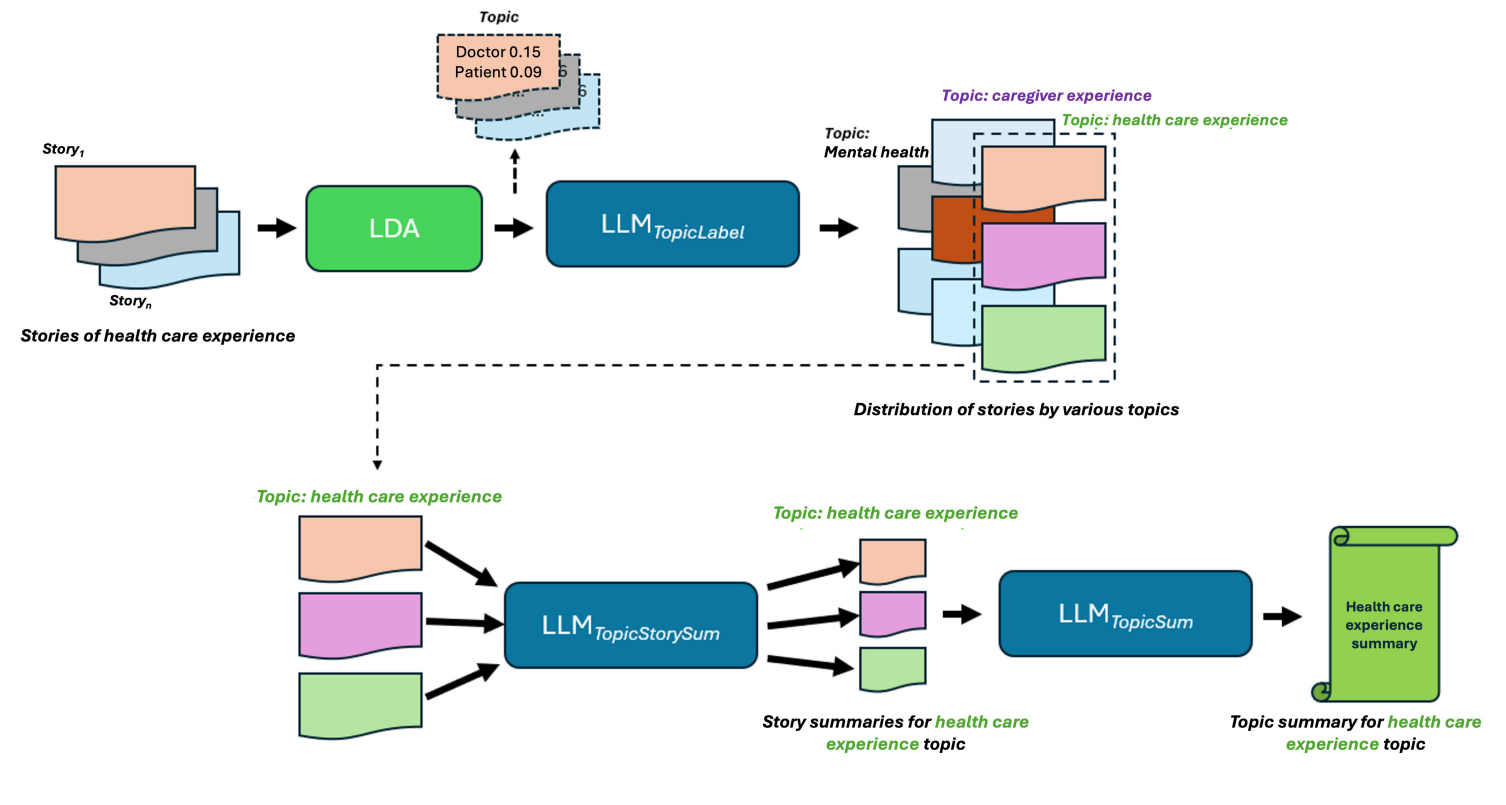
Various steps in the proposed approach for a topic-aware large language model (LLM) for long-form spoken-dialogue summarization. LDA: latent Dirichlet allocation.

The method for topic-aware summarization of long-form health care experiences combined classical methods of topic detection with recent advances in LLMs through a multistep process. First, topic detection was performed using the LDA approach [42]. The identified topics were then labeled for interpretability using LLMs, and topic story summaries were generated. Finally, topic labels and story-topic distributions from the LDA were leveraged for topic-aware summarization using a hierarchical summarization approach powered by LLMs. Individual steps are further described in the *Topic Detection and Labeling* and *Hierarchical Summarization* sections. Figure 1 shows various components of the proposed approach.

#### Topic Detection and Labeling

To understand recurring topics from health care experience stories, it was necessary to identify the topics across stories. Topic modeling approaches such as LDA are often the go-to solutions for such problems and are widely used in studying health care experiences, as discussed in the related work. LDA is a probabilistic topic modeling method that captures the likelihood of topics across documents (here, stories) and simultaneously the distribution of words in each topic. LDA was used to detect topics across all 1120 stories from the dataset. The topic detection model was tuned by varying the number of topics between 50 and 1000 in steps of 50. The optimal number of topics was identified using a perplexity criterion on our validation set.

The word distributions in each topic offered a naive understanding of topics. They presented the likelihood of words in each topic but were often hard to interpret. They might also contain contradictory, ambiguous, or unrelated words under the same topic. This issue was more prevalent in the dataset, considering that the participant perspectives included patients, caregivers, and health care professionals, each offering a variety of perspectives, which made understanding the conversational themes more challenging. To overcome this limitation in interpretation, word clouds of topics were labeled using a pretrained Llama-3.1 model [43]. The Llama is an open-source LLM with competitive performance against closed-source alternatives such as GPT-4 [44] and GPT-3.5 Turbo [45]. For all LLM experiments, the 70B parameter model was used due to its long context length of 128K tokens. The inference time was optimized using the *llama.cpp* [46] framework with 4-bit quantization and GPT-Generated Unified Format encoding.

Given the challenging nature of the task, labels for each topic were derived within the context of each story. The LLM input consisted of a story and the word list from the topic-word distribution (Multimedia Appendix 1). The words were ranked in the order of their likelihood in the topic as determined by the LDA. Topic labels derived in the context of a story tend to overfit to a specific story. Moreover, across multiple stories, the topic label was found to be paraphrases of one another. To establish the consistency of topic labels across all relevant stories, the most frequent label of the topic across all stories was chosen as the final topic label.

#### Hierarchical Summarization

**Figure 2.**
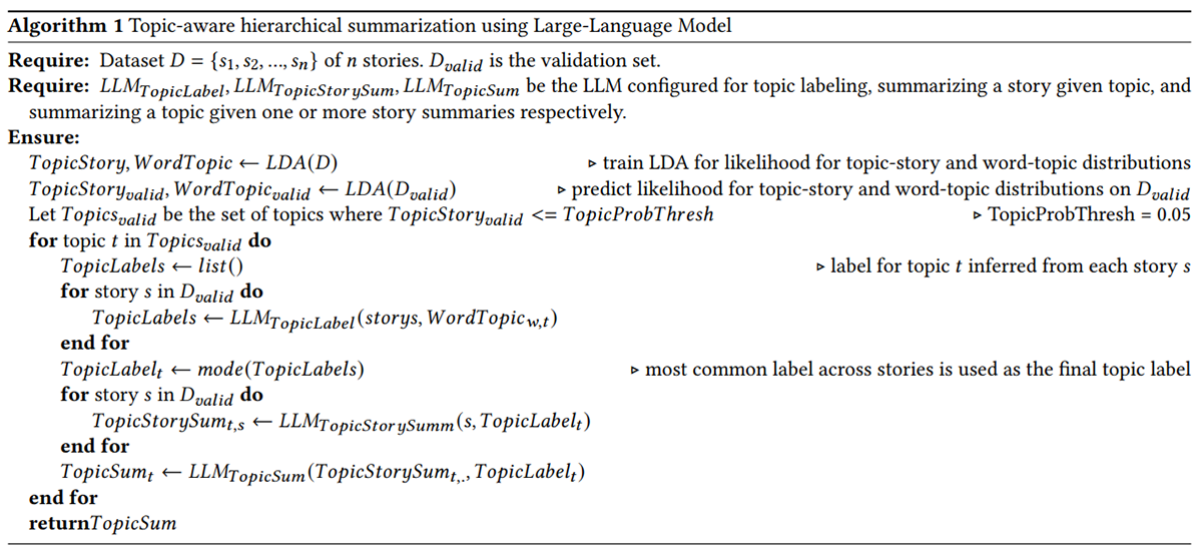
Topic-aware hierarchical summarization using large language models (LLMs). LDA: latent Dirichlet allocation.

Figure 2 presents a step-by-step overview of the approach. The LDA-identified topics were leveraged to summarize the stories from the validation set. To overcome the input context length limitations, a hierarchical summarization approach was used. The hierarchical summarization approach involved 2 steps. In the first step, topic summarization for each story was performed (Multimedia Appendix 1). This greatly consolidated the input and overcame the limitation of input context length for long-form summarization with LLMs. Following this, individual topic story summaries were further summarized to generate a holistic summary of all the stories under the topic (Multimedia Appendix 1). The hierarchical summarization also offered interpretability through tracing elements of the holistic summary.

#### Evaluation

A strong evaluation of generative models was critical to understanding their limitations. Conventional evaluation metrics, such as ROUGE (Recall-Oriented Understudy for Gisting Evaluation) [47], only account for co-occurrence similarity between generated and reference text. Hence, in the context of LLMs, these similarity-based metrics make little sense. LLM-generated content is also prone to a distinct set of challenges, such as hallucinations and inadvertent safety limitations, which are not studied using such conventional metrics. To account for these shortcomings, we used the multidimensional evaluation framework developed with a focus on the quality of information, understanding and reasoning, expression style and persona, safety and harm, and trust and confidence principles, referred to as QUEST [48], to evaluate the generated summaries. The evaluation dimensions for this summarization task include comprehensiveness, fabrication, accuracy, and usefulness.

Human evaluation was often expensive and time-consuming, so the LLM’s ability to generate topic summaries was assessed using the LLM-as-a-judge approach. To eliminate self-enhancement bias in LLMs, GPT-4 Turbo was used as the judge. GPT-4 was found to correlate well with human raters and was less susceptible to position bias [49]. Individual topic summaries, together with the respective topic story summaries, were used as input to rate the 4 QUEST dimensions on a 5-point Likert scale (see Table S1 in Multimedia Appendix 2 for the definition of our Likert scale). The reliability of the GPT responses was quantified against 2 domain experts as human raters. Raters were assigned 4 topics (chronic pain management, medical treatment, caregiving experience, and health concerns) to rate the QUEST dimensions on the same Likert scale as the GPT model. Discrepancies in Likert values between the 2 human raters were discussed and adjudicated by those raters to provide a final rating, which was compared to the GPT responses. The Bennett *S*-score [50] was used with quadratic weighting to calculate agreement between the raters and the GPT responses.

## Results

### Topic Detection and Labeling

The LDA model yielded 150 topics on the training set. For the validation set, we inferred document-level topic distributions and applied a threshold on topic probabilities to identify highly relevant topics for each story and to avoid spurious assignments. For stories where the topic probability did not exceed the threshold, the most likely topic was assigned. This approach allowed us to minimize the adverse downstream effects in the hierarchical summarization pipeline due to incoherent topic assignment. Using this approach, 40 topics were identified in the validation set.

Topic labels for the LDA topics were necessary for the topic-aware summarization approach. Such labels also improved topic interpretability for domain experts. As mentioned in the *Methods* section, the topic labeling approach using LLM resulted in 26 topic labels across all the stories (see Table 1 for topic labels, where the numbers in parentheses indicate the number of stories that address each topic), while the remaining 14 topics were omitted due to the inherent difficulty in labeling topics based on the distribution of words and redundancy among the labels. These topic labels were then used in the summarization step.

**Table 1.** Topic labels derived from the large language model (LLM).

Topic labels derived from the large language model	Number of stories that address the topic
Health care experience	(38)
Healthy eating	(13)
Doctor-patient relationship	(11)
Cancer treatment	(10)
Hospital experience	(9)
Doctor experience	(6)
Personal experience	(5)
Disease diagnosis	(3)
Health care	(3)
Caregiving	(2)
Cancer diagnosis	(1)
Emotional and physical impact	(1)
Heart health	(1)
Symptoms and hospitalization	(1)
Health care system	(26)
Caregiving experience	(11)
Symptom management	(11)
Chronic pain management	(10)
Surgical experience	(7)
Medical treatment	(6)
Health concerns	(3)
Mental health	(3)
Diagnosis	(2)
Diabetes management	(1)
Health challenges	(1)
Medical condition	(1)

### Hierarchical Summarization

The topic labels, together with the corresponding stories, were used to generate topic summaries through the intermediate topic story summaries. The results of hierarchical summarization for 2 sample topics are provided in Table 2 (see Table S1 in Multimedia Appendix 3 for summaries from corresponding topics). We found that the summaries for each topic were comprehensive, coherent, and aligned with the topic label derived based on the LDA topic-word distribution.

**Table 2.** Sample summaries for 2 different topics from our hierarchical summarization approach.

Topic label	Topic summary
Chronic pain management	Participants dealing with chronic pain management reported various challenges and experiences. Many struggled with inadequate pain management, including ineffective treatments, lack of effective pain medication, and hesitation from health care providers to treat their pain. Some participants felt that their pain was not taken seriously, leading to frustration and increased severity. Others found alternative methods, such as cannabidiol oil, yoga, and herbs, to be helpful in managing their pain. A few participants benefited from working with supportive doctors, pain clinics, and transportation programs that helped them access necessary care. Despite these efforts, many participants continued to experience chronic pain, numbness, and debilitating symptoms, affecting their daily lives and relationships.
Caregiver experience	Caregivers face numerous challenges, including lack of support, financial struggles, and emotional toll. Many participants expressed the importance of compassion, patience, and affection when caring for loved ones, particularly those with dementia or Alzheimer disease. Some participants had to navigate complex health care systems, advocate for their loved ones’ needs, and deal with the emotional impact of caregiving. Others appreciated the support of family members, health care providers, and online support groups. Several participants highlighted the need for respite care, financial assistance, and guidance for caregivers. Some also emphasized the importance of acknowledging stress and seeking help. Additionally, participants noted that health care providers should advise patients on healthy living and provide resources for caregivers. Overall, caregiving experiences varied, but many participants shared the common theme of needing more support and resources to effectively care for their loved ones.

### Evaluation

We evaluated the generated summaries alongside topic story summaries to perform a human evaluation. Table 3 presents the interrater and GPT-rater agreements derived from our evaluation approach. A moderate-to-high agreement was noticed between the GPT responses and the raters. The level of agreement was particularly strong for the fabrication and comprehensiveness of the generated summaries. This suggested that the GPT evaluation of generated summaries overlapped with human raters and could be used reliably to evaluate the topic-aware hierarchical summarization model. GPT responses for all topics included in the validation set are provided in Table S2 in Multimedia Appendix 2. The accuracy of the generated summaries was consistently good for most topics, except for *diagnosis,* where the generated summary likely changed the interpretation of the topic because of one substantive point within it. The usefulness of the summaries was found to be mostly high, indicating that the approach was promising to use in place of manually identifying themes in the story and summarizing them, as defined by our Likert scale for evaluation. Although these findings were based on 4 topics, together they covered up to 30 stories from the validation set of 50 stories across the 4 QUEST dimensions, thus allowing for diversity both in terms of stories and assessment criteria.

**Table 3.** Bennett *S*-score agreement between raters and GPT averaged over 4 topics after adjudication^a^.

QUEST^b^ dimension	*S*-score (R1, R2)	*S*-score (GPT, R1)	*S*-score (GPT, R2)	∑_i_*S*-score (GPT, R_i_)/2
Fabrication	0.94	0.94	1.00	0.97
Accuracy	0.81	0.62	0.69	0.65
Comprehensiveness	0.94	0.94	0.87	0.91
Usefulness	1.00	0.75	0.75	0.75

a The score ranges from −1 to 1, where −1, 0, and 1 indicate perfect disagreement, chance-level agreement, and perfect agreement, respectively. *S*-score (A, B) denotes the agreement between raters A and B.

b QUEST: quality of information, understanding and reasoning, expression style and persona, safety and harm, and trust and confidence.

In addition to the ratings from experts, their free-form comments suggested that our transcription and summarization processes were error-prone. Errors, such as incorrect dosage units when referring to medication intake (“I take *30 kilos* of medication a day. I used to take *50 kilos* a day”), were due to difficulties and challenges in our Whisper-based transcription approach where “30 pills” was incorrectly transcribed as “30 kilos.” Lexical inconsistencies when referring to a single story (“*Many* participants expressed the importance of compassion, patience…” was actually expressed by only 1 person), deviations from the topic of interest, and a lack of cohesion in topic with fewer stories can also be observed.

## Discussion

### Principal Contributions and Findings

We introduce a computational method using automated transcription, topic modeling, and summarization to extract insights from narratives of health care experiences. It was found that automated approaches provide quality transcriptions when validated against manual transcriptions, and topic modeling identified 26 topics. Some common topics from our dataset include health care experiences, healthy eating, caregiving, doctor-patient relationships, symptom management, cancer treatment, and chronic pain management. Through our summarization approach, we expand the scope of understanding health care experiences beyond the conventional coarse sentiment analysis [12,13,32] to offer details related to clinical interactions, treatment efficacy, and their outcomes from individual narratives. Generated summaries, when assessed using the LLM-as-a-judge framework, were found to be free from fabrication, highly accurate, comprehensive, and useful. LLM assessments of generated summaries concur with expert findings. Our hierarchical summarization approach is particularly useful for extracting such insights from large narrative datasets. The summaries for all 26 topics identified from the narratives can be found in Table S2 in Multimedia Appendix 4.

### Topic Detection and Labeling

An important component of our proposed approach is topic modeling using LDA. Among the 150 topics identified in the training set, 40 were present in the validation set. This difference in topic coverage between the training and validation sets could be due to 2 factors. First, the validation set constitutes only about 5% of the training set. Thus, several topics that were observed in the training set may not be sufficiently captured in the validation set. Alternatively, our topic probability threshold prioritized topic relevance over coverage to limit any adverse effect on the downstream hierarchical summarization due to incoherent topic assignments. Interestingly, only 26 out of 40 topics can be labeled for topic names using the LLM. This difficulty in topic labeling was also observed during the early attempts to manually assign topic labels using the topic distribution of words by domain experts. This suggests that both humans and LLMs find it challenging to attribute an interpretable topic label from the high-dimensional representation of topics and words. Despite the modest number of topics, redundancy among the topics is noticeable. Topics such as *health care experience and health care system, caregiving experience and caregiving, and doctor-patient relationship and doctor experience* are closely related. The authors would like to highlight that such redundancy in topics is an expected outcome of LDA and the choice of hyperparameters; addressing it remains an active area of research in NLP [51]. In addition, this redundancy hinders interpretability for domain experts in designing interventions. We believe ontology-based approaches, such as the graph-sparse LDA [52] or semantic LDA [53], could be explored. Such approaches leverage the structure of the vocabulary to enforce sparsity or capture semantic meaning, which limits such redundancy and improves interpretability. While LDA is a popular choice for topic modeling, it assumes a Dirichlet distribution of topics in a document. Alternatives, such as probabilistic latent semantic indexing [54], may result in different topics. The authors would also like to highlight that, in addition to probabilistic generative approaches, such as LDA and probabilistic latent semantic indexing, the NLP community has also begun exploring LLMs for topic modeling [55,56].

### Hierarchical Summarization and Evaluation

The summarization step leverages the superior summarization capabilities of LLMs to offer additional insights into the narratives. The hierarchical summarization overcomes the challenge of the limited input context of LLMs by generating individual topic story summaries, followed by topic summaries for corresponding stories. The evaluation framework addresses the limitations of conventional metrics for summarization, such as ROUGE, by using a multidimensional approach derived from the QUEST framework. It was found that GPT-4 Turbo evaluations concur with human evaluations, and the summaries provided by our approach are comprehensive, accurate, free of fabrication, and useful.

Human evaluation is often expensive and time-consuming. Here, we found that LLMs can help to summarize story contents, identifying several relevant insights and key topics in an efficient and timely manner. However, tradeoffs are involved, such as considerable topic redundancy and uncertainty regarding accuracy in summary details. Maintaining a human element in the evaluation process can help to identify and address such concerns.

### Limitations and Future Work

This work also has some limitations. The automatic transcription was evaluated against only a small set of human transcriptions. Findings suggest that minor but critical errors in transcription, such as misinterpreting units of medication (*kilos* for *pills*), propagate to the final summary. Early manual intervention to correct transcription errors, including those due to differences in speaker accents, linguistics, and environmental variables, through a larger dataset remains a future endeavor. Such manual intervention could prevent error propagation to later stages of the pipeline. Recent work [55,56] studied the efficacy of LLMs and prompting techniques on topic modeling, finding that LLMs can identify topics and offer explainability. We believe that this direction may help address the topic redundancy and lack of explainability limitations of conventional topic models such as LDA. The hierarchical summarization uses a 70B parameter Llama LLM due to its demonstrated task generalization performance (eg, 82% in reading comprehension, 93% in math reasoning, and 85% in common sense understanding) and emergent capabilities [57]. However, the rapidly evolving LLM landscape warrants a comparison between alternative choices, such as Qwen [58] and DeepSeek [59], to help identify the optimal model for summarization. The GPT-based approach for summarization evaluation was limited to comparing topic summaries with individual story-topic summaries rather than comparing the topic summaries against the original transcripts. This assumes that the generated story-topic summaries are sufficiently comprehensive, accurate, and free from any fabrication. Future work shall incorporate the human evaluation of generated topic summaries through comparison with the original stories from each topic to better assess summary accuracy. The quantitative measures of agreement in human evaluation should be interpreted while keeping the limited focus on 4 topics in perspective. The small dataset (50 stories) limits the topic detection and summarization outcomes. However, it reflects a design choice put in place to enable a future analysis directly comparing LLM and traditional qualitative evaluations. Future work shall consider larger datasets collected from different geographical locations and health care infrastructures for a better understanding of experiences. Large-scale datasets could offer greater insights into the health care experience through improved topic coverage and diversity. It is also possible that summarizing larger datasets may lead to an increase in the risk of hallucinations and reduced factuality due to the lack of access to a comprehensive ontology that spans across the health care ecosystem. Evaluating such large-scale systems requires rater agreements to be studied on a large number and variety of topics. However, when selecting topics, care should be taken to avoid the risk of rater fatigue, as a given summary may require assessing tens or hundreds of its constituent stories. Pipeline-based approaches, such as our topic detection followed by hierarchical summarization, are brittle due to error propagation between successive steps (as observed with the transcription error). End-to-end approaches overcome this limitation; we believe that exploring end-to-end approaches by integrating topic modeling together with summarization is another exciting direction for future research.

### Conclusions

This work contributes to the health care informatics domain using NLP techniques (topic detection and hierarchical summarization) to offer an understanding of narratives of health care experiences beyond the conventional sentiment analysis approach, which is popular in existing literature on health care experiences. We leverage both traditional and modern NLP techniques, such as LDA and LLMs, to achieve this goal. Using widely accepted quantitative metrics for reliability, it was demonstrated that crucial steps in the proposed approach (such as transcription of audio recordings of the narratives and evaluation of summaries) can be performed with sufficient reliability using recent advances in speech processing and LLMs. We believe that this provides an opportunity to attract interest from the community and accelerate research using narratives to improve health care experiences and health equity through computational methods.

## Supplementary material

10.2196/85960 Multimedia Appendix 1 Prompt templates used with large language models (LLMs) for topic labeling and hierarchical summarization.

10.2196/85960 Multimedia Appendix 2 Likert-scale definitions and GPT responses to QUEST (quality of information, understanding and reasoning, expression style and persona, safety and harm, and trust and confidence principles) dimensions used in the LLM-as-a-judge evaluation.

10.2196/85960 Multimedia Appendix 3 Topic story summaries used to generate sample topic summaries.

10.2196/85960 Multimedia Appendix 4 Generated summaries for each of the 26 topics from the validation set.

## Abbreviations

LDA: latent Dirichlet allocation
LLM: large language model
NLP: natural language processing
QUEST: quality of information, understanding and reasoning, expression style and persona, safety and harm, and trust and confidence
ROUGE: Recall-Oriented Understudy for Gisting Evaluation
