# Supplementary material for: Topic-Aware Summarization of Lived Health Care Experiences: Large Language Model Evaluation Study
Source: JMIR Med Inform. 2026 Jun 11;14:e85960. doi: 10.2196/85960 (PMC13258062; doi:10.2196/85960)
Supplement: Multimedia Appendix 1 [file medinform-v14-e85960-s001.docx]

**Multimedia Appendix 1:** Prompt templates used with LLMs for topic labeling and hierarchical summarization.

1. Topic Labeling Prompt

[SYSTEM]

Given the participant experience dealing with the healthcare system, identify the topic labels that fit the experience based on the given list of topic words. Each list should correspond to only one topic label. Given output in <TOPIC LABEL>: LIST OF WORDS format. No additional text is required in the output.

[USER]

Participant experience: <STORY>

List of topic words: <WORDS IN STORY>

2. Topic Story Summarization Prompt

[SYSTEM]

Summarize the experience of participant dealing with the healthcare system along the given topic. Use participant(s) own words during summarization and do not paraphrase.

[USER]

Participant(s) experience: <EXPERIENCE>

Topic label: <TOPIC LABEL>

3. Topic Summarization Prompt

[SYSTEM]

Generate a holistic summary experience from individual participant(s) summaries about dealing with the healthcare system along the given topic. Note that all summaries should fit and not deviate from the topic label. Do not include any additional information apart from that present in the input. Do not paraphrase.

[USER]

Participant(s) summaries: <PARTICIPANT STORY SUMMARIES>

Topic label: <TOPIC LABEL>
