# Supplementary material for: Topic-Aware Summarization of Lived Health Care Experiences: Large Language Model Evaluation Study
Source: JMIR Med Inform. 2026 Jun 11;14:e85960. doi: 10.2196/85960 (PMC13258062; doi:10.2196/85960)
Supplement: Multimedia Appendix 2 [file medinform-v14-e85960-s002.docx]

**Multimedia Appendix 2: Likert-scale definitions and GPT responses to QUEST dimensions used in the LLM-as-a-judge evaluation**.

**Table S1:** Likert-scale definition used for QUEST dimensions used in the GPT-based and human evaluation of topic summaries.

| **QUEST dimension and definition** | **Likert-scale definition** |
| --- | --- |
| **Fabrication** (The summary contains made-up information or data that is not covered in the story. This includes any plausible but non-existent facts**)** | 1.       Wholly fabricated.  2.       Largely fabricated.  3.       One substantive point fabricated – i.e., the fabrication changes the meaning/interpretation of the topic of interest.  4.       One minor detail fabricated – i.e., fabrication is present but does not change the meaning/interpretation of the topic of interest.  5.       No made-up information. |
| **Accuracy** (The summary is factually correct, precise, and free from any errors) | 1. Wholly inaccurate.  2. Largely inaccurate.  3. One substantive point is inaccurate (i.e., inaccuracy changes the interpretation of the topic of interest).  4. One minor detail is inaccurate (i.e., inaccuracy is present but does not change the interpretation of the topic of interest).  5. No inaccuracies in the summary. |
| **Comprehensiveness** (The summary covers all critical themes (i.e., patient concerns about healthcare experience) discussed in the story. It offers a completely comprehensive overview of the story along with sufficient details) | 1. Topic summary covers none of the critical themes from the story summaries.  2. Topic summary is missing most of the critical themes from the story summaries.  3. One substantive theme is missing from the summary (which shifts the interpretation of the topic of interest).  4. One minor theme is missing from the summary (which does not impact interpretation of the topic of interest).  5. All critical themes are covered in the topic summary. |
| **Usefulness** (The summary is useful. It can be reliably used in place of manually identifying key themes in the story and then summarizing them) | 1. Topic summary cannot be used at all in place of manually identifying key themes from the story summaries.  2. Topic summary is largely not useful for identifying key themes from the story summaries.  3. Topic summary has a moderate degree of usefulness for identifying key themes from the story summaries.  4. Topic summary is mostly useful for identifying key themes from the story summaries.  5. The topic summary is extremely useful for identifying key themes from the story summaries. |

**Table S2:** GPT-4-Turbo responses to evaluating topic summaries on a 5-point Likert scale. The Likert-scale definition can be found in Multimedia Appendix 2 Table S1.

| **Topic** | **Fabrication** | **Accuracy** | **Comprehensiveness** | **Usefulness** |
| --- | --- | --- | --- | --- |
| **Doctor Experience** | 5 | 5 | 5 | 5 |
| **Medical Condition** | 5 | 5 | 5 | 5 |
| **Healthcare Experience** | 5 | 5 | 5 | 5 |
| **Chronic Pain Management** | 5 | 4 | 4 | 5 |
| **Hospital Experience** | 5 | 5 | 5 | 5 |
| **Surgical Experience** | 5 | 5 | 5 | 5 |
| **Caregiving Experience** | 5 | 5 | 5 | 5 |
| **Healthcare System** | 5 | 5 | 5 | 5 |
| **Symptom Management** | 5 | 5 | 4 | 5 |
| **Medical Treatment** | 5 | 5 | 4 | 4 |
| **Health Challenges** | 5 | 5 | 4 | 4 |
| **Healthy Eating** | 5 | 5 | 5 | 5 |
| **Diabetes Management** | 5 | 5 | 5 | 5 |
| **Doctor-Patient Relationship** | 5 | 4 | 4 | 5 |
| **Health concerns** | 5 | 5 | 5 | 5 |
| **Cancer Treatment** | 5 | 5 | 4 | 5 |
| **Caregiving** | 5 | 5 | 4 | 5 |
| **Personal Experience** | 5 | 5 | 4 | 4 |
| **Diagnosis** | 5 | 3 | 4 | 4 |
| **Healthcare** | 5 | 5 | 4 | 4 |
| **Disease Diagnosis** | 5 | 5 | 5 | 5 |
| **Mental Health** | 5 | 5 | 5 | 5 |
| **Cancer Diagnosis** | 5 | 5 | 5 | 5 |
| **Heart Health** | 5 | 5 | 5 | 5 |
| **Symptoms and Hospitalization** | 5 | 5 | 5 | 5 |
| **Emotional and Physical Impact** | 5 | 5 | 5 | 5 |
