# Supplementary material for: Topic-Aware Summarization of Lived Health Care Experiences: Large Language Model Evaluation Study
Source: JMIR Med Inform. 2026 Jun 11;14:e85960. doi: 10.2196/85960 (PMC13258062; doi:10.2196/85960)
Supplement: Multimedia Appendix 3 [file medinform-v14-e85960-s003.docx]

**Multimedia Appendix 3:** Topic **s**tory summaries used to generate sample topic summaries.

**Table S1:** Topic **s**tory summaries used to generate topic summaries for samples used in table 2. Each paragraph is a separate story summary for the topic.

| **Topic label** | **Topic story summary** |
| --- | --- |
| Chronic Pain Management | I'm still dealing with chronic pain and numbness in my hand after the injury. I've seen a pain doctor and had nerve blockers, but the pain persists. I'm frustrated with the lack of effective pain management and the need for repeated surgeries and procedures.     The participant struggled with chronic pain due to endometriosis and experienced debilitating symptoms, including heavy bleeding, clots, and nausea. They felt that their pain was not taken seriously by some healthcare providers and were not offered adequate pain management options.     I have been using CBD oil to manage my chronic pain as conventional over-the-counter treatments don't work for me, and I don't want a pain prescription. On really bad days, I use an ultra-sized tampon or a menstrual cup to help manage my symptoms.     I was in a lot of pain for a long time, with sharp pains in my stomach and leg. I had to try different pain meds, but they didn't work. I was always in bed and lost my drive. After surgery, I felt better, but I had to learn how to manage my stress and not let it affect my relationships with my kids.     The participant's aunt suffered from chronic pain due to her diabetes and amputations, but was not provided with adequate pain management, leading to further suffering and decline in her health.     I've been dealing with an injury from 20 years ago that's causing me chronic pain. Despite this, I've been sent to physical therapy, which I don't think will fix the underlying problem. I feel like the healthcare system is trying to avoid giving me the treatment I need, specifically an operation, and instead is trying to manage my pain with temporary solutions.     I have chronic pain and I take 30 kilos of medication a day. I used to take 50 kilos a day, but I've been able to reduce my medication. I have a chronic pain doctor who helps me manage my pain. I also have a transportation program that helps me get to my doctor's appointments, which is free.     The participant struggled with chronic pain management, particularly with regards to their arm and hand. They experienced numbness, tingling, and pain, which made everyday activities challenging. They tried various treatments, including medication, physical therapy, and hand therapy. They also participated in a pain clinic, which helped them learn coping mechanisms and understand how pain affects behavior. They found that the pain clinic was a turning point in their management of chronic pain and that it helped them develop a better understanding of their condition.     I've learned to take care of myself and put myself in positions where I'm not stressing about unnecessary things. I've also had to relearn my body and figure out what triggers my lupus. My doctors have been helpful in managing my pain, and I've had infusions and other treatments to maintain my condition.     Managing chronic pain is a challenge. I've had to deal with hesitation from healthcare providers to treat my pain, which can lead to longer hospital stays and more severe pain. However, I've found that working with a supportive doctor and using holistic approaches like yoga and herbs can help manage my pain. |
| Caregiver Experience | The participant's family members, including their mother and grandmother, took on caregiving roles for their niece and grandmother, respectively. The participant expressed concerns about the lack of support for caregivers, including respite care and financial assistance, and the impact on their quality of life.     The participant's experience with caregiving was shaped by her young age and stressful circumstances during her pregnancy. She had to navigate abusive relationships, financial struggles, and poor eating habits, which she believes may have impacted her son's health. Despite these challenges, she was grateful for the support of her family and a particular doctor who took the time to ask about her lifestyle and well-being. She learned the importance of acknowledging stress and seeking help, and wishes that she had received more guidance and support as a young mother.     I don't currently have a caregiver, but I try to stay independent and take care of myself. I also try to support others who are caregivers or patients, and I participate in online support groups to connect with others who share similar experiences.     The participant shares their experience of being a caretaker to their mother who was diagnosed with dementia Alzheimer's. They emphasize the importance of being compassionate, patient, and affectionate, and accepting the patient's reality. They also highlight the need to involve the patient in activities, allow them to maintain their independence, and show love and support. The participant notes that it's essential to be hands-on, listen to the patient, and validate their feelings, even if it means hearing repeated stories. They also stress the importance of not showing anger or frustration, even when the patient becomes aggressive or accusatory.     The participant's experience as a caregiver was emotionally challenging, and they felt that the caregiving system failed to provide adequate support and resources. They had to navigate complex healthcare systems and advocate for their aunt's needs, but ultimately felt that they were not equipped to provide the level of care she required.     The participant had a challenging experience with aftercare, as they were not able to get a caregiver due to insurance limitations. They had to rely on family members and church members for support, and eventually had to find their own way to manage their care. They suggest that healthcare providers should advise patients on healthy living and provide resources for caregivers.     The participant became a caregiver for their father when he was diagnosed with prostate cancer. They had to navigate the healthcare system to get him the care he needed and had to fight to get him tested for the BRCA gene. They also had to deal with the emotional toll of caring for a loved one and eventually had to make the decision to put him in hospice care.     The participant's sister played a significant caregiving role, driving to the hospital and providing support during the recovery process. The participant also appreciated the hospital's arrangement for a nurse to visit their home, ensuring their safety and well-being as they lived alone.     The participant's experience as a certified nursing assistant (CNA) is highlighted, where they took care of elderly residents in nursing homes, assisting with daily living activities such as bathing, feeding, and dressing. They also shared a memorable experience of caring for an AIDS patient who was initially resistant to care but eventually warmed up to them.     I took care of my mother for many years, managing her medical appointments and caring for her needs. It was a challenging experience, especially as she became more depressed and withdrawn. I also had help from my family, including my husband and children, when I was recovering from my car accident in 1965.     The participant's adoptive mother was often absent and neglectful, leaving them and their disabled older brother alone for long stretches of time. The participant had to rely on a charity organization and state-funded counselors for support, but felt that their concerns were not taken seriously. |
