# Supplementary material for: Topic-Aware Summarization of Lived Health Care Experiences: Large Language Model Evaluation Study
Source: JMIR Med Inform. 2026 Jun 11;14:e85960. doi: 10.2196/85960 (PMC13258062; doi:10.2196/85960)
Supplement: Multimedia Appendix 4 [file medinform-v14-e85960-s004.docx]

**Multimedia Appendix 4:** Generated summaries for each of the 26 topics from the validation set.

**Table S2:** Generated summaries for each of the 26 topics identified from the 50 stories of healthcare experience from African American individuals.

| **Topic** | **Topic summary** |
| --- | --- |
| Healthy Eating | Many participants emphasized the importance of healthy eating in managing their health conditions, such as pre-diabetes, type 2 diabetes, hypertension, cancer, and sickle cell disease. However, some participants faced challenges in making healthy food choices, including lack of understanding, limited access to healthy food options, and difficulty changing old eating habits. Some participants had to research and figure out healthy eating habits on their own, while others worked with healthcare professionals, such as nurse practitioners, nutritional counselors, and dietitians, to develop personalized eating plans. Common themes included the importance of eating a balanced diet, avoiding processed and sugary foods, and staying hydrated. Some participants also noted the importance of considering individual lifestyle and food preferences when making healthy eating choices. Overall, participants recognized the significance of healthy eating in managing their health conditions and improving their overall well-being. |
| Chronic Pain Management | Participants dealing with chronic pain management reported various challenges and experiences. Many struggled with inadequate pain management, including ineffective treatments, lack of effective pain medication, and hesitation from healthcare providers to treat their pain. Some participants felt that their pain was not taken seriously, leading to frustration and further suffering. Others found alternative methods, such as CBD oil, yoga, and herbs, to be helpful in managing their pain. A few participants benefited from working with supportive doctors, pain clinics, and transportation programs that helped them access necessary care. Despite these efforts, many participants continued to experience chronic pain, numbness, and debilitating symptoms, affecting their daily lives and relationships. |
| Doctor Experience | Some participants had negative experiences with doctors, feeling dismissed, talked down to, and disrespected. Doctors were perceived as arrogant, uncaring, and unwilling to consider alternative explanations or provide second opinions. In contrast, other participants had positive experiences with doctors who were supportive, listened to their concerns, and provided alternatives and explanations. These doctors were described as wonderful, rock stars, and understanding, taking the time to get to know the participants and their goals. |
| Emotional and Physical Impact | The participant experienced a prolonged period of pain and stress, compounded by the emotional toll of their mother's death and caregiving responsibilities. They continue to suffer from aching and numbness in their leg, necessitating ongoing pain management with medication. |
| Hospital Experience | Participants had varied experiences with hospitals, ranging from disappointment and frustration to relief and gratitude. Some participants felt ignored, like a nuisance, or moved through like cattle, while others appreciated empathetic and supportive healthcare providers. Efficient intake and diagnosis were noted by some, while others experienced challenges with communication and disconnection from healthcare providers. Traumatic experiences, including life-threatening episodes and prolonged stays in critical care, were also reported. However, some participants were grateful for the care they received and appreciated the efforts of hospital staff. Regular hospital visits for ongoing treatments, such as blood transfusions, were also mentioned, highlighting the emotional toll of diagnosis and treatment. |
| Disease Diagnosis | Participants' experiences with disease diagnosis highlight the challenges and importance of seeking professional help. One participant with early-onset Alzheimer's and breast cancer emphasizes the need for proactive health management. Another participant with cardiac sarcoidosis experienced a delayed diagnosis, taking five years to confirm, and wished for earlier diagnosis to better understand and manage their care. A third participant with MDS initially felt shock and denial but eventually became an advocate for themselves and others after coming to terms with their condition. |
| Cancer Diagnosis | Dealing with the healthcare system after a cancer diagnosis can be challenging, particularly when insurance coverage is disputed. One individual's experience involved a recent job change and new insurance, which initially refused to cover their cancer treatment, citing a pre-existing condition despite the diagnosis occurring just two weeks after starting the job. This led to a prolonged fight with the insurance company to secure coverage for necessary medical procedures. |
| Caregiving | Caregiving experiences were marked by feelings of unpreparedness and insufficient support. Participants took on caregiving roles for family members with serious health conditions, such as end-stage renal failure, and struggled to balance caregiving responsibilities with work and personal life. Despite receiving some help from home health agencies and family members, participants faced difficulties in providing 24-hour care and managing increasing care needs. The caregiving system was perceived as failing to provide adequate support, leading to feelings of regret and frustration when participants were unable to continue caring for their loved ones. |
| Symptom Management | Managing symptoms is a daily challenge for many participants, who struggle with chronic pain, heavy bleeding, nausea, and other issues. Some feel that their healthcare providers do not take their symptoms seriously, leading to frustration and a need for self-advocacy. Participants have found various ways to manage their symptoms, including medication, self-care, and lifestyle changes. Some have learned to plan their day around their medication and make adjustments to their diet to minimize symptoms. Others have found alternative treatments, such as photo light therapy, and have learned to prioritize stress management and self-care. Despite the challenges, many participants have found ways to persevere and take care of themselves, often with the support of family, doctors, and other healthcare professionals. |
| Cancer Treatment | Treatment Approaches and Outcomes    Some participants were able to manage their cancer through surgery, exercise, and early detection, without requiring chemotherapy, radiation, or medication.  Others underwent various treatments, including chemotherapy, radiation, mastectomy, and reconstructive surgery, with some experiencing side effects and complications.  One participant also incorporated alternative therapies, such as acupuncture and Chinese herbs, into their treatment plan.  One participant underwent standard treatment for colon cancer, including chemotherapy and genetic testing, and took preventive measures to reduce their risk of developing other types of cancer.    Decision-Making and Patient Autonomy    Some participants reported making informed decisions about their treatment plans, including opting for mastectomy or seeking second opinions.  One Participant was proud of their decision to opt for a mastectomy instead of chemotherapy and radiation, given their HIV-positive status.  One Participant felt that they were not given the option to have genetic testing for the BRCA gene early on, which may have changed their treatment plan.    Emotional and Psychological Impact  Some participants emphasized the importance of staying positive, focusing on what they can do, and managing their emotional well-being during and after treatment.  One participant  highlighted the emotional impact of being diagnosed with a life-threatening condition, even if it is not cancer.    Importance of Early Detection and Patient Engagement    Some participants stressed the importance of early detection and managing one's own health in achieving successful treatment outcomes.  Participant  suggested that patients should listen to their doctors, take notes, and ask questions to ensure they understand their treatment plan. |
| Diagnosis | Lung cancer diagnosis was unexpected for both participants, with no prior symptoms. Routine doctor's visits and chest x-rays revealed tumors on the upper left lobe of the lung. One participant's diagnosis was prompted by a mention of struggling to reach certain notes while singing, while the other had a routine check-up. Doctors' confidence and thorough explanations helped alleviate initial fear. |
| Health Challenges | Dealing with the healthcare system is a significant challenge due to the complexity of multiple health conditions, including thalassemia minor, diabetes, heart disease, arthritis, scleritis, trigger fingers, tendonitis, corporal tunnel, high blood pressure, asthma, vocal cord dysphonia, and thyroid issues, which collectively impact daily life. |
| Medical Treatment | Participants experienced mixed results with medical treatment, with some encountering misdiagnosis, delayed treatment, and unhelpful guidance. Others found relief with healthcare providers who took a holistic approach, listened attentively, and used evidence-based methods. Challenges included traveling for specialized treatment, dealing with medical errors, and affording expensive medication. Some participants faced difficulties in finding effective treatments for chronic conditions like PCOS, endometriosis, and rheumatoid arthritis. |
| Doctor-Patient Relationship | Participants had varied experiences with their doctors, ranging from positive to negative. Some appreciated doctors who were approachable, willing to simplify medical terms, and listened to their concerns. Others had negative experiences with doctors who were rushed, condescending, dismissive, or unwilling to listen. Some participants valued the importance of a good rapport with their doctor, citing the need for respect, clear communication, and a willingness to work together. A few participants appreciated doctors who were knowledgeable about specific communities, such as the LGBTQ+ community. Some participants also highlighted the importance of being able to ask questions and receive clear explanations about their condition and treatment options. Overall, participants emphasized the need for doctors to be empathetic, communicative, and responsive to their needs. |
| Heart Health | Managing heart health involves a complex and challenging experience, requiring learning to manage the condition through medication and lifestyle changes, emphasizing the importance of listening to one's body and communicating with doctors for optimal care. |
| Healthcare | Participants have reported negative experiences with the healthcare system, citing issues with government-assisted healthcare, such as long wait times, packed waiting rooms, and doctors who are not aware of patients' backgrounds and needs. Healthcare professionals have also been criticized for not respecting patients' time, rushing appointments, and lacking communication skills. Additionally, some participants have experienced insensitive treatment from healthcare providers, including being asked insensitive questions, being dismissed, and not being listened to, resulting in inadequate care. |
| Surgical Experience | Participants had varied experiences with surgical procedures, ranging from traumatic to positive. Some participants experienced anxiety, pain, and complications, such as infections, implant issues, and unhealed wounds. Others had successful surgeries with quick recoveries. Support from healthcare providers was inconsistent, with some participants receiving empathetic care and others feeling dismissed or unsupported. |
| Diabetes Management | Dealing with the healthcare system for diabetes management involves struggling to find the right support until meeting a specialized nurse practitioner who provides necessary guidance. This support includes understanding the diagnosis, developing a treatment plan, and making lifestyle changes that improve health. Additionally, it involves learning the importance of self-advocacy and seeking specialist care. Ultimately, it leads to empowerment to educate others about effective diabetes management and the value of supportive care. |
| Health Concerns | Managing multiple health issues is a common challenge, as seen in the experiences of these participants. They face difficulties in keeping track of medications and appointments while dealing with various health concerns such as heart problems, knee problems, low blood pressure, cancer, thyroid problems, high blood pressure, arthritis, and MDS. These conditions significantly impact their daily lives, causing symptoms like pain, swelling, fatigue, dizziness, and shortness of breath. The participants have tried various medications, but they often only provide temporary relief, leading to concerns about the risk of stroke and the high cost of medication, which can be as high as $32,000 a month. |
| Medical Condition | Dealing with Reflex Sympathetic Dystrophy (RSD), a chronic pain condition affecting the nervous system, was a challenging experience. Severe pain, swelling, and limited mobility in the knee were not adequately addressed by the initial doctor, requiring a second opinion. A specialist confirmed the diagnosis, but treatment was complicated by the workers' compensation system, limiting access to care and medication. |
| Mental Health | Participants have experienced various mental health issues, including anxiety, depression, PTSD, dissociative disorder, chronic depression, insomnia, and CPTSD, which have been exacerbated by difficulties in the healthcare system, stigma, and traumatic experiences. They have faced challenges such as feeling dismissed, long waitlists, uneducated providers, and inadequate care. Some have relied on self-researched coping mechanisms, while others have had to try multiple therapists and medications to find what works for them. |
| Healthcare Experience | The participants' experiences with the healthcare system are varied, with some reporting positive encounters and others facing challenges and frustrations. Common themes include:    1. Lack of empathy and understanding: Many participants felt disrespected, dismissed, or not taken seriously by healthcare providers, leading to feelings of frustration and mistrust.  2. Difficulty navigating the system: Participants often struggled to find the right doctors, get referrals, and access necessary treatments, highlighting the complexity and fragmentation of the healthcare system.  3. Challenges with insurance and affordability: Several participants faced issues with insurance coverage, medication costs, and affordability, which affected their ability to access necessary care.  4. Importance of self-advocacy: Many participants emphasized the need to take an active role in their own healthcare, researching their conditions, questioning doctors, and seeking second opinions to ensure they receive the best care possible.  5. Positive experiences with supportive providers: Some participants reported positive experiences with healthcare providers who were empathetic, knowledgeable, and willing to work with them to manage their conditions.  6. Challenges with chronic conditions: Participants with chronic conditions, such as diabetes, cancer, and mental health issues, often faced difficulties in managing their conditions and accessing necessary treatments.  7. Impact of stigma and bias: Some participants reported experiencing stigma and bias due to their conditions, such as HIV, addiction, or sickle cell disease, which affected their interactions with healthcare providers.  8. Importance of support systems: Participants often highlighted the importance of having a support system, including family, friends, and support groups, to help navigate the healthcare system and manage their conditions.    Overall, the participants' experiences highlight the need for a more patient-centered, empathetic, and supportive healthcare system that addresses the complex needs of individuals with various conditions. |
| Personal Experience | Living with chronic conditions and navigating the healthcare system can be a challenging and isolating experience. Participants have had to deal with severe side effects, stigma, and discrimination, while also learning to advocate for themselves and others. Despite these challenges, many have found ways to take care of themselves, such as eating healthy, researching, and trying new things. Supportive networks, including family and friends, have also played a crucial role in their journeys. Participants have learned to appreciate the preciousness of life and the importance of living in the moment, and have found ways to grow and self-discover through their experiences. |
| Healthcare System | The participants' experiences with the healthcare system are overwhelmingly negative, with many expressing frustration, disappointment, and feelings of neglect. Common themes include:    1. Lack of support and understanding from healthcare providers, particularly for chronic conditions, mental health, and minority communities.  2. Inadequate communication, empathy, and compassion from healthcare providers.  3. Difficulty navigating the healthcare system, including accessing specialized care, transportation, and services.  4. Insufficient education and awareness about various health conditions, including HIV, AIDS, and rare diseases.  5. Perceived bias and discrimination within the healthcare system, particularly towards minority communities and women.  6. Need for more emphasis on patient empowerment, self-advocacy, and personalized care.  7. Importance of addressing the root causes of health problems, rather than just treating symptoms.  8. Frustration with the lack of research and funding for certain health conditions.  9. Difficulty accessing affordable care and medication, particularly for those with limited resources or support.  10. Need for better standards and regulations for caregiving companies and support for caregivers and their clients.    Overall, the participants' experiences highlight the need for a more patient-centered, compassionate, and equitable healthcare system that addresses the unique needs and challenges of diverse populations. |
| Caregiving Experience | Caregivers face numerous challenges, including lack of support, financial struggles, and emotional toll. Many participants expressed the importance of compassion, patience, and affection when caring for loved ones, particularly those with dementia or Alzheimer's. Some participants had to navigate complex healthcare systems, advocate for their loved ones' needs, and deal with the emotional impact of caregiving. Others appreciated the support of family members, healthcare providers, and online support groups. Several participants highlighted the need for respite care, financial assistance, and guidance for caregivers. Some also emphasized the importance of acknowledging stress and seeking help. Additionally, participants noted that healthcare providers should advise patients on healthy living and provide resources for caregivers. Overall, caregiving experiences varied, but many participants shared the common theme of needing more support and resources to effectively care for their loved ones. |
| Symptoms and Hospitalization | Initial misdiagnosis of nasal congestion led to hospitalization due to breathing difficulties and chest tightness. A series of tests, including blood tests and a kidney biopsy, resulted in a diagnosis of lupus. Repeated hospital visits were necessary to drain fluid from lungs and manage symptoms. |
